# Immediate word recall in cognitive assessment can predict dementia using machine learning techniques

**DOI:** 10.1186/s13195-023-01250-5

**Published:** 2023-06-15

**Authors:** Michael Adebisi Fayemiwo, Toluwase Ayobami Olowookere, Oluwabunmi Omobolanle Olaniyan, Theresa Omolayo Ojewumi, Idowu Sunday Oyetade, Shannon Freeman, Piper Jackson

**Affiliations:** 1 Department of Computer Science, Redeemer’s University, Ede, Osun State Nigeria; 2 School of Nursing, University of Northern British Columbia, Prince George, British Columbia Canada; 3 School of Computing, Engineering and Intelligent Systems, Ulster University, Londonderry, Northern Ireland UK; 4 Department of Computing Science, Thompson Rivers University, Kamloops, British Columbia Canada

**Keywords:** Cognitive assessment, Dementia, Machine learning, Medical data analytics, Word recall

## Abstract

**Background:**

Dementia, one of the fastest-growing public health problems, is a cognitive disorder known to increase in prevalence as age increases. Several approaches had been used to predict dementia, especially in building machine learning (ML) models. However, previous research showed that most models developed had high accuracies, and they suffered from considerably low sensitivities. The authors discovered that the nature and the scope of the data used in this study had not been explored to predict dementia based on cognitive assessment using ML techniques. Therefore, we hypothesized that using word-recall cognitive features could help develop models for the prediction of dementia through ML techniques and emphasized assessing the models’ sensitivity performance.

**Methods:**

Nine distinct experiments were conducted to determine which responses from either sample person (SP)’s or proxy’s responses in the “word-delay,” “tell-words-you-can-recall,” and “immediate-word-recall” tasks are essential in the prediction of dementia cases, and to what extent the combination of the SP’s or proxy’s responses can be helpful in the prediction of dementia. Four ML algorithms (K-nearest neighbors (KNN), decision tree, random forest, and artificial neural networks (ANN)) were used in all the experiments to build predictive models using data from the National Health and Aging Trends Study (NHATS).

**Results:**

In the first scenario of experiments using “word-delay” cognitive assessment, the highest sensitivity (0.60) was obtained from combining the responses from both SP and proxies trained KNN, random forest, and ANN models. Also, in the second scenario of experiments using the “tell-words-you-can-recall” cognitive assessment, the highest sensitivity (0.60) was obtained by combining the responses from both SP and proxies trained KNN model. From the third set of experiments performed in this study on the use of “Word-recall” cognitive assessment, it was equally discovered that the use of combined responses from both SP and proxies trained models gave the highest sensitivity of 1.00 (as obtained from all the four models).

**Conclusion:**

It can be concluded that the combination of responses in a word recall task as obtained from the SP and proxies in the dementia study (based on the NHATS dataset) is clinically useful in predicting dementia cases. Also, the use of “word-delay” and “tell-words-you-can-recall” cannot reliably predict dementia as they resulted in poor performances in all the developed models, as shown in all the experiments. However, immediate-word recall is reliable in predicting dementia, as seen in all the experiments. This, therefore, shows the significance of immediate-word-recall cognitive assessment in predicting dementia and the efficiency of combining responses from both SP and proxies in the immediate-word-recall task.

## Background

According to the Lancet [1] dementia is identified as one of the fastest-growing public health problems. Dementia is a condition that results from several diseases and affects memory, cognitive abilities, and behavior that interfere significantly with a person’s ability to maintain their activities of daily living. Dementia is not a natural aspect of growing older, even though age is the most vital recognized risk factor [2–4]. Globally, about 50 million people have dementia, and there are nearly 10 million new cases yearly [5]. A prevalent type of dementia is Alzheimer’s disease which may contribute to 60–70% of cases [5]. The impact of dementia on caregivers, family, and society can be physical, psychological, social, and economic. This could result in stigmatization, causing people to live in self-denial which ultimately constitutes barriers to diagnosis and care. Moreover, it could lead to dependency among older people, which can be overwhelming for caregivers [6]. Dementia affects a person in different ways depending on the impact of the disease and the person’s personality before becoming ill. Signs and symptoms related to dementia are generally in the early, middle, and late phases. It can be challenging to recognize the early signs and symptoms of dementia which are often disregarded since the development can be gradual. Awareness of signs and symptoms may increase during the middle phase as they are more noticeable, prominent, and limiting than those in the early stage [7]. In the intermediate stage, the people may become increasingly confused, lost, and absent-minded about current events and names, have difficulty communicating with others, need assistance with private care, and exhibit behavioral changes [5, 8]. The late stage shows near-total dependence and inactivity, difficulty recognizing relatives and friends, and behavior changes that may lead to aggression [9, 10]. According to the 2017 report of the Lancet Commissions [11], acting on dementia prevention, intervention, and care early enough would aid the recovery of individuals with dementia enormously, as it is the most significant global challenge for health and social care in the 21st century [11]. Currently, there is no cure for dementia, but several treatments are being investigated in various stages of clinical trials [5, 12]. The early diagnosis of dementia is vital for the treatment and care of patients [13, 14]. Diagnosis of dementia commonly requires wide-ranging evaluation like reviewing of the medical history, mental state, and cognitive function evaluation, clinical laboratory testing, neuropsychological testing, evaluation of daily-living activities, and brain imaging testing [9, 15] which are some of the critical areas where ML applications are used in the healthcare. However, ML application in healthcare is complex and challenging [16], especially in diagnosis and prediction [17]. The use of ML approach and deep learning (DL) algorithms are used in diagnosing and prognosis of dementia, using the clinical status of an individual and/or brain imaging testing datasets [9, 18–23].

Jammeh [24] conducted a feasibility study on ML to identify undiagnosed dementia in healthcare. The study was born out of the hypothesis that states the possibility of identifying undetected dementia from myriads of symptoms. The implementation was carried out using a read-encoded dataset regularly gathered from healthcare services. Read codes are a collection of medical terminologies employed to condense medical and organizational data for public practice in the United Kingdom. The read-encoded data collected from 26,483 participants aged 65 years and above was utilized for the implementation [24]. The authors verified read codes given to participants who may be responsible for an individual having dementia. These codes were used to train an ML prediction model to identify people who have primary dementia. The 15,469 read codes collected from the 26,483 patients comprise 6,101 medication codes, 4301 diagnosis codes, and 5028 care codes. The medication codes represent any medication that may have been recommended. The diagnosis codes keep the diagnosis log, whereas the process of care codes keeps track of symptoms, history, tests, examinations, etc. The study used random forest, Support vector machine, logistic regression, and Naive Bayes algorithms as classifiers while employing specificity, AUC, accuracy, and sensitivity. It was reported that the Naive Bayes classifier gave the best performance with a sensitivity and specificity of 84.47% and 86.67%, respectively [24].

To construct an ML-based predictive model for future cognitive impairment, Na [25] used a gradient-boosting machine. The study population consisted of 3,424 community elders free of cognitive impairment, gotten from the dataset from the Korean Longitudinal Study of Aging (KLoSA). The study classified elders at risk for cognitive impairment after 2 years. It was discovered that the discovered number of elders with cognitive impairment after the 2 years was 80 (2.34%). The predictive model with gradient boosting machine showed sensitivity, AUC, specificity, and accuracy values of 0.968, 0.921, 0.825, and 0.829, respectively. The precision-recall plot of their model showed that the model performed well irrespective of the use of the highly imbalanced dataset. The results also established that age, Mini-Mental Status Examination (MMSE), and education levels contributed to the predictive model.

Zhu [23] developed a tool for clinicians to diagnose the early stage of dementia. The model was able to identify normal cognition, mild cognitive impairment, very mild dementia, and dementia using an informant-based 37-item questionnaire filled by 5272 individuals. Three different feature selection methods including random forest, information gain, and relief algorithms were used to select the best features, and it was discovered that information gain was the most effective. The set features were then fed into six classification models. The Naive Bayes model had excellent accuracy, precision, recall, and F-measure values of 81%, 82%, 81%, and 81%, respectively. The model was able to identify normal cognition with a sensitivity of 84% and specificity of 94%, respectively. For mild cognitive impairment identification, the model achieved a sensitivity of 62% and specificity of 93%, and for very mild dementia, the results are sensitivity of 72% and specificity of 93%. For dementia, the sensitivity of 92% and specificity of 95% were recorded, respectively.

Casanova [26] used ML to examine predictors of the cognitive decline of dementia. Random forest analyzed modifiable and hereditary risk factors for Alzheimer's disease to detect cognitive decline. The results gave 78%, 75%, and 81% accuracy, sensitivity, and specificity values. Their technique revealed that the top-ranking predictors are education, diabetes, APOE ε4 carrier status, age, gender, stroke, NSES, and body mass index. Non-genetic factors contribute the most to cognitive decline than genetic factors, according to the study, and education is the most significant contributor to cognitive discrimination.

Kim and Lim [9] developed DNN/scaled PCA, a technique based on a deep neural network for predicting dementia, utilizing extensive data. This study used data from 7,031 participants over 65 years old from the Korea National Health and Nutrition Examination Survey (KNHANES) in 2001 and 2005 to train a deep neural network to predict dementia using health-related behavior and healthcare service consumption data. The model employed principal component analysis and min/max scaling to preprocess and extract necessary background attributes. The study compared their developed methodology, a deep neural network/scaled principal component analysis, with random forest, AdaBoost, multilayer perceptron, Gaussian Naive Bayes, and SVM. The developed methodology showed 85.5% of the AUC, which was better than all other machine learning algorithms. The sensitivity, specificity, precision, accuracy, and AUC values that resulted from the DNN method were 68.6%, 82.1%, 5.4%, 81.9%, and 85.5%, respectively.

Di [27] used realistic driving data and preliminary findings from the longitudinal research on aging drivers (LongROAD) study to detect moderate cognitive impairment and dementia. The Longitudinal Research on Aging Drivers collected driving data from 2977 volunteers every month using in-vehicle recording equipment lasting up to 45 months. This yielded 29 variables that measured space, driving behavior, and efficiency. The random forests algorithm was utilized to categorize the participant's moderate cognitive impairment/dementia level during the follow-up. Random forests’ F1-score in discriminating mild cognitive impairment/dementia level was 29% based on demographical character traits alone (age, sex, race/ethnicity, and education), 66% based on driving variables alone, and 88% based on demographical character traits and driving variables. Feature importance analysis revealed that age, sex, race/ethnicity, education, and driving variables were the most predictive features of mild cognitive impairment and dementia with accuracy, precision, sensitivity, specificity, and AUC of 86%, 86%, 90%, 81%, and 90% respectively.

Velazquez and Lee [28] employed random forest to predict individualized mild cognitive impairment to Alzheimer’s disease conversion. The study used four ML algorithms (random forest, support vector machine, logistic regression, and XGBoost) to model dementia prediction on three sets of selected features (6, 9, and 13 features). The study showed that the random forest model had the best performance regarding nine features with accuracy, precision, sensitivity, and AUC of 93.6%, 95.2%, 97.8%, and 96%.

### Objectives

From the findings reviewed, it was discovered that though most of the models developed had high accuracies, some, however, suffer from relatively low sensitivities. An accuracy score alone cannot be used as a reliable measure in a delicate health application domain such as dementia prediction. The predictive performances of sensitivity (depicting the predictive accuracy of the dementia class) in the models built on most of the datasets used in literature have been considerably low, as seen in Casanova [26] Zhu [23] Kim and Lim [9], and Di [27] However, higher sensitivity scores have been recorded in Na [25] and Velazquez and Lee [28] The clinical importance of the above-highlighted models based on the reported sensitivity to the dementia class can, however, be improved upon. Therefore, the primary objective of this study is to improve the predictive performances (especially sensitivity) of dementia predictive models based on word-recall cognitive assessment using machine learning techniques. In light of this, the study sets out to answer the following specific research questions:

1. Can responses from SP solely help to predict dementia?
2. Can responses from proxies solely help to predict dementia?
3. Can the combination of responses from both SP and proxies help predict dementia?
4. Which word-recall cognitive assessment contributes best to the prediction of dementia cases?
5. To what extent can a machine learning model be used to predict cases of dementia using features from Word-recall cognitive assessment?

## Methods

### Source of data

The dataset used in this study was obtained from the National Health and Aging Trends Study database [29]. NHATS is a one-of-a-kind national resource for scientific research on later-life health, which started in 2011. The goal of NHATS is to encourage research that will help lead initiatives to lessen disability, improve well-being and independence, and improve the quality of life in older people. NHATS collects data from a nationally representative sample of Medicare beneficiaries in persons 65 years of age and older to accomplish its goals. In the supplementary National Study of Caregiving (NSOC), carers of NHATS participants were occasionally interviewed.

### Participants and sample size

The data gathered by the National Health and Aging Trends Study (NHATS) [29] was drawn from a nationally representative survey of Medicare recipients between the ages of 65 and older. Interviews with carers of NHATS respondents in the supplementary National Study of Caregiving (NSOC) were conducted on an occasional basis. The Johns Hopkins University Bloomberg School of Public Health and the University of Michigan's Institute for Social Research are in charge of NHATS and NSOC, while Westat is in charge of data collection. Round 8 of the data collection has a dataset that contains 5547 rows and 1317 columns and has four types of respondents named; sample persons (4679), proxy (664), inapplicable (92), and missing (112), for which cognitive assessment features are stated among others, for dementia. A search on PubMed from inception to 15 October 2021 for studies investigating dementia prediction with machine learning on cognitive features using the NHATS data was performed via the use of title terms “dementia” AND “prediction” AND “machine learning” AND “cognitive assessment” AND “NHATS”, combined with title/abstract terms or MeSH terms “dementia prediction” AND “machine learning” AND “cognitive assessment” AND “National Health Aging Trends Study” with no limits on language or date of publication. We found no paper that had employed the use of machine learning techniques for predicting dementia with cognitive features using the NHATS dataset at that time.

### Study design

The study was co-developed by a team of artificial intelligence researchers and health experts from Redeemer’s University, Ede, Nigeria; Thompson Rivers University, British Columbia, Canada; and University of Northern British Columbia, Canada.

**Fig. 1:**
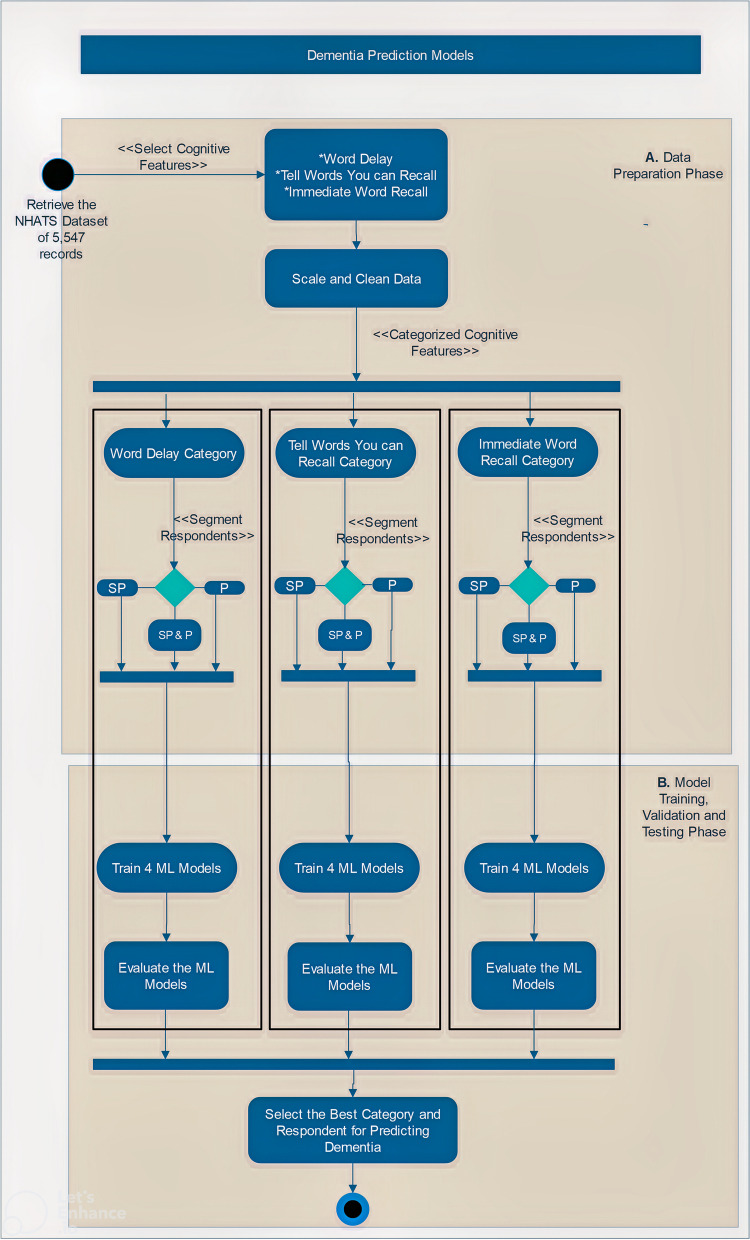
The workflow of the study. SP, sample person; P, proxy; SP&P, sample person and proxy; ML, machine learning; NHATS, National Health and Aging Trends Study

A standardized dataset on dementia from the National Health and Aging Trends Study (NHATS) was obtained from the organization’s public data bank [29] to carry out this study. NHATS is sponsored by the National Institute on Aging (grant number NIA U01AG032947) through a cooperative agreement with the Johns Hopkins Bloomberg School of Public Health [30]. To construct ML predictive models to predict dementia from the obtained dataset, four conventional classification ML algorithms, including K-nearest neighbor (KNN), decision tree (DT), random forest (RF), and artificial neural networks (ANN), were adopted. The purpose of selecting these algorithms is not to determine which ML model has the highest potential for the prediction of dementia; instead, it is to examine through the use of the ML models which cognitive assessment method(s) can best predict cases of dementia. An overview of the workflow of the study is illustrated in Fig. 1. As shown in the designed workflow, cognitive features are selected from the NHSAT dataset.

### Predictors

The extracted features categories include “word-delay” cognitive assessment task, “tell-words-you-can-recall” cognitive assessment task, “Immediate Words Recall” cognitive assessment task. Three distinct respondents were objectively segmented for each cognitive assessment task category for analysis. These respondents include SP, proxies, and the combination of SP and proxies.

### Development and validation

The data corresponding to each respondent in the individual cognitive assessment task scenario was then presented to the four ML algorithms to train dementia predictive models. Consequently, the study carried out the training of nine (9) distinct ML models. After that, an objective evaluation of the trained ML models was carried out for each model trained for each respondent and in each scenario. The ML performance evaluation metrics considered for evaluating these models include accuracy, 10-fold cross-validation accuracy, precision, F1 score, and specificity, emphasizing the sensitivity metric. Based on these performance evaluation processes, the best respondent concerning the best scenario in predicting dementia with high sensitivity was then selected as a standard cognitive assessment for dementia prediction. In this study, the cut-off point on the ROC curve was selected by using the approach that maximizes the sensitivity, in which a threshold that maximizes the true positive rate (TPR) while keeping the false positive rate (FPR) below beneath was determined. The obtained best threshold for maximizing sensitivity while keeping the false positive rate below was 0.83.

Experimental models’ development and analyses were performed using Python on the Google Collaboratory (Google Colab) environment. Google Colab provided us with Python 3 Google compute engine backend (TPU) used to train all models.

The Python 3 Google compute engine backend has all the required latest versions of Python packages and libraries installed, including but not limited to the following: NumPy, matplotlib, pandas, yellow-brick, sklearn, pandas, and Keras, while pyreadstat and scikitplot were manually installed on the environment. The Python codes for all the experiments carried out in this study can be found at https://doi.org/10.6084/m9.figshare.16817506.v3 [31].

In this research, NHATS Round 8 dataset was used. The dataset is publicly available data at https://nhats.org/researcher/data-access/public-use-files. The dataset contains 5547 rows and 1317 columns and has four types of respondents named; SP (4679), proxy (664), inapplicable (92), and missing (112). According to Kasper and Freedman [32], a maximum number of SP responded to the interview for themselves, with proxy responders utilized where the SP could not reply. When a proxy respondent was used, data was collected based on the following factors: (i) the reason for the proxy's use (either dementia or cognitive impairment reported by proxy, the sample person was too ill, speech or hearing impairment, communication barriers, or sample person was inaccessible); (ii) the proxy's relationship to the sample person; and (iii) proxy familiarity with the sample person's everyday routine. In this study, the outcomes obtained from the use of these respondents (sample person and proxy) will answer research questions 1 to 3.

The cognition section of the NHATS dataset is intended to give information regarding some parts of cognitive functioning, which includes memory (self-rated, whether memory affects day-to-day activities, and immediate and delayed 10-word-recall), orientation (date, month, year, day of the week; naming President and Vice President), and executive function (clock drawing test) [32]. This study only focuses on word-recall using “word-delay,” “tell-words-you-can-recall,” and “immediate-word-recall.”

This study performed nine experiments with the use of different respondents (sample person, proxy, or combination of sample person and proxy responses). In proxy responses, 468 sample persons had No Dementia while 196 sample persons had Dementia. In sample person responses, 4481 SP had “No Dementia” while 464 SP had “Dementia”. Proxy and sample person responses were combined using OR operator on “Dementia” and AND operator on “No Dementia”. For the combination of proxy and sample person responses, 445 sample persons had “No Dementia” while 491 sample persons had “Dementia”. These experiments were further divided into three different scenarios; “word delay”, “tell words you can recall”, and “immediate word recall”. The feature list details for these scenarios can be found in the Appendix.

## Results

### Model development

This study carried out nine experiments, comprising three separate experiment scenarios, each in four scenarios. The first scenario of experiments concerns the use of the “word-delay” feature in predicting dementia, while the second scenario of experiments involves the use of the “tell the words you can recall” feature in predicting dementia. The third scenario of experiments uses the “immediate-word-recall” feature in predicting dementia. Each of these experiments was performed based on the SP’s responses, the proxy’s responses, and the combination of the SP’s and proxy’s responses in each of the three cognitive assessment tasks considered.

The classification performance of the four ML models trained in the highlighted experiments for predicting dementia is compared and shown in Tables 1, 2, and 3. The accuracy, cross-validation accuracy, sensitivity, specificity, precision, and F1 measure of each of the considered models are all reported.

**Table 1:** Classification report of the classifiers in the “word delay” cognitive assessment task (scenario 1)

	**Cross-validation accuracy (%)**	**Accuracy (on data split) (%)**	**Precision**		**f1-score**	***Sensitivity***	***Specificity***
			***Dementia***	***Normal***			
**A. Scenario 1A:** on the sample person’s responses							
**KNN**	92.00	93.00	0.74	0.94	0.92	0.37	0.99
**Decision Tree**	91.00	92.00	0.55	0.94	0.91	0.37	0.97
**Random forest**	92.00	92.00	0.59	0.94	0.91	0.38	0.97
**Artificial neural networks**	92.00	93.00	0.72	0.94	0.92	0.37	0.99
**B. Scenario 1B:** on the proxy’s responses							
**KNN**	72.00	69.00	0.47	0.71	0.62	0.11	0.94
**Decision tree**	72.00	69.00	0.50	0.71	0.62	0.10	0.96
**Random forest**	72.00	69.00	0.50	0.71	0.62	0.10	0.96
**Artificial neural networks**	72.00	69.00	0.00	0.69	0.57	0.00	1.00
**C. Scenario 1C:** on the sample person’s and proxy’s responses							
**KNN**	77.00	78.00	0.96	0.70	0.77	0.60	0.97
**Decision tree**	77.00	78.00	0.96	0.69	0.77	0.59	0.97
**Random forest**	77.00	78.00	0.96	0.70	0.77	0.60	0.97
**Artificial neural networks**	77.00	78.00	0.96	0.70	0.77	0.60	0.97

**Table 2:** Classification report of the classifiers in the “tell words you can recall” task (scenario 2)

	**Cross-validation accuracy (%)**	**Accuracy (on data split) (%)**	**Precision**		**f1-score**	***Sensitivity***	***Specificity***
			***Dementia***	***Normal***			
**A. Scenario 2A:** on the sample person’s responses							
**KNN**	92.00	93.00	0.74	0.94	0.92	0.37	0.99
**Decision tree**	90.00	92.00	0.53	0.94	0.91	0.42	0.96
**Random forest**	92.00	93.00	0.62	0.94	0.92	0.42	0.98
**Artificial neural networks**	92.00	93.00	0.65	0.95	0.92	0.44	0.98
**B. Scenario 2B:** on the proxy’s responses							
**KNN**	71.00	69.00	0.50	0.70	0.58	0.02	0.99
**Decision tree**	72.00	69.00	0.50	0.71	0.61	0.08	0.96
**Random forest**	72.00	69.00	0.50	0.71	0.62	0.10	0.96
**Artificial neural networks**	70.00	70.00	0.75	0.70	0.60	0.05	0.99
**C. Scenario 2C:** on the sample person’s and proxy’s responses							
**KNN**	77.00	78.00	0.96	0.70	0.77	0.60	0.97
**Decision tree**	77.00	76.00	0.95	0.68	0.75	0.56	0.97
**Random forest**	77.00	78.00	0.96	0.69	0.77	0.59	0.97
**Artificial neural networks**	76.00	78.00	0.96	0.69	0.77	0.59	0.97

**Table 3:** Classification report of the classifiers in the “immediate word recall” cognitive assessment task (scenario 3)

	**Cross-validation accuracy (%)**	**Accuracy (on data split) (%)**	**Precision**		**f1-score**	***Sensitivity***	***Specificity***
			***Dementia***	***Normal***			
**A. Scenario 3A:** on the sample person’s responses							
**KNN**	93.00	93.00	0.77	0.94	0.92	0.36	0.99
**Decision tree**	93.00	93.00	0.77	0.94	0.92	0.36	0.99
**Random forest**	93.00	93.00	0.77	0.94	0.92	0.36	0.99
**Artificial neural networks**	92.00	93.00	0.73	0.94	0.92	0.37	0.99
**B. Scenario 3B:** on the proxy’s responses							
**KNN**	88.00	91.00	0.79	0.98	0.91	0.97	0.88
**Decision tree**	88.00	92.00	0.80	0.99	0.92	0.98	0.89
**Random forest**	88.00	92.00	0.80	0.99	0.92	0.98	0.89
**Artificial neural networks**	88.00	91.00	0.83	0.95	0.91	0.89	0.92
**C. Scenario 3C:** on the sample person’s and proxy’s responses							
**KNN**	95.00	94.00	0.89	1.00	0.94	**1.00**	0.88
**Decision tree**	95.00	94.00	0.89	1.00	0.94	**1.00**	0.88
**Random forest**	95.00	94.00	0.89	1.00	0.94	**1.00**	0.88
**Artificial neural networks**	93.00	94.00	0.89	1.00	0.94	**1.00**	0.88

#### Experiments scenario 1: using “word-delay” feature

This scenario of experiments focuses on using the “word-delay” feature in predicting dementia. Each section of the NHATS data that specify the responses of SP and proxy’s responses to the “word-delay” cognitive assessment was extracted and split into a training set (70%) and a test set (30%). Each of the four models (KNN, RF, DT, and ANN) was trained on the training set, and dementia prediction was done on the test set.

- *Experiment scenario 1A:* This experiment was carried out using only the SP’s responses to a “word-delay” cognitive assessment task to model and predict dementia.
- *Experiment scenario 1B:* This experiment was carried out using only the proxy’s responses to a “word-delay” cognitive assessment task to model and predict dementia.
- *Experiment scenario 1C:* This experiment was carried out using the combination of the SP’s and proxy’s responses to a “word-delay” cognitive assessment task to model and predict dementia.

For these experiments, Table 1 shows the models’ performances in terms of accuracy, cross-validation accuracy, precision, F1 score, sensitivity, and specificity.

**Fig. 2:**
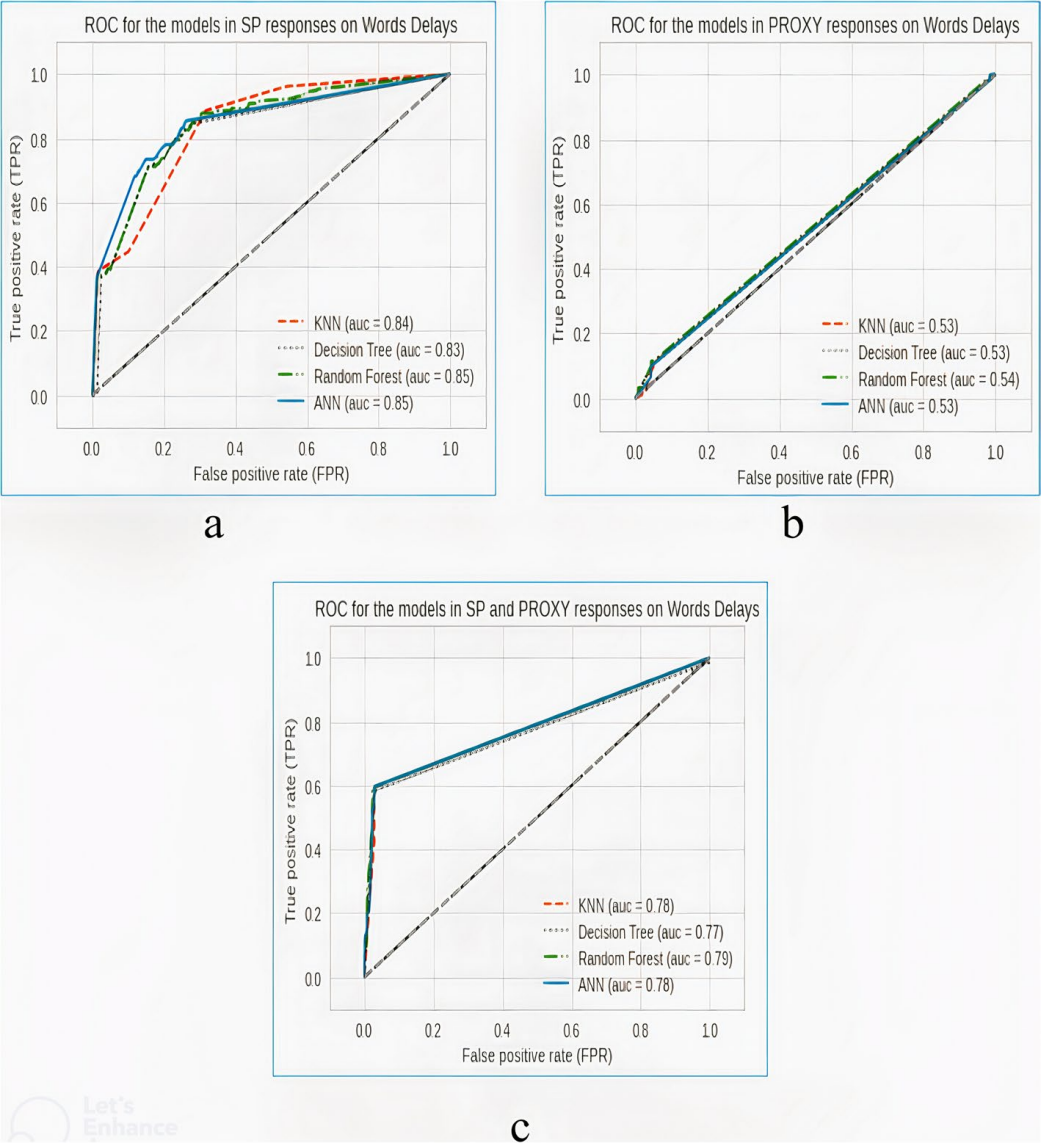
Receiver’s operating characteristics curve obtained from the models in a “word-delay” task based on **a** SP’s responses, **b** proxy’s responses, and **c** a combination of the SP’s and proxy’s responses

Figure 2 shows the receiver’s operating characteristics curves obtained from the models in experiments scenarios 1A, 1B, and 1C.

#### Experiments scenario 2: using “tell-words-you-can-recall” feature

This scenario of experiments focuses on using the “tell-words-you-can-recall” feature in predicting dementia. Each section of the NHATS data that specify the responses of SP and proxy’s responses to the “tell-words-you-can-recall” cognitive assessment was extracted and split into a training set (70%) and a test set (30%). Each of the four models (KNN, RF DT, and ANN) was trained on the training set, and dementia prediction was done on the test.

- *Experiment scenario 2A:* This experiment was carried out using only the SP’s responses to a “tell-words-you-can-recall” cognitive assessment task to model and predict dementia.
- *Experiment scenario 2B:* This experiment was carried out using only the proxy’s responses to a “tell-words-you-can-recall” cognitive assessment task to model and predict dementia.
- *Experiment scenario 2C:* This experiment was carried out using the combination of the SP’s and proxy’s responses to a “tell-words-you-can-recall” cognitive assessment task to model and predict dementia.

For these experiments, Table 2 shows the models’ performances in terms of accuracy, cross-validation accuracy, precision, F1 score, sensitivity, and specificity.

**Fig. 3:**
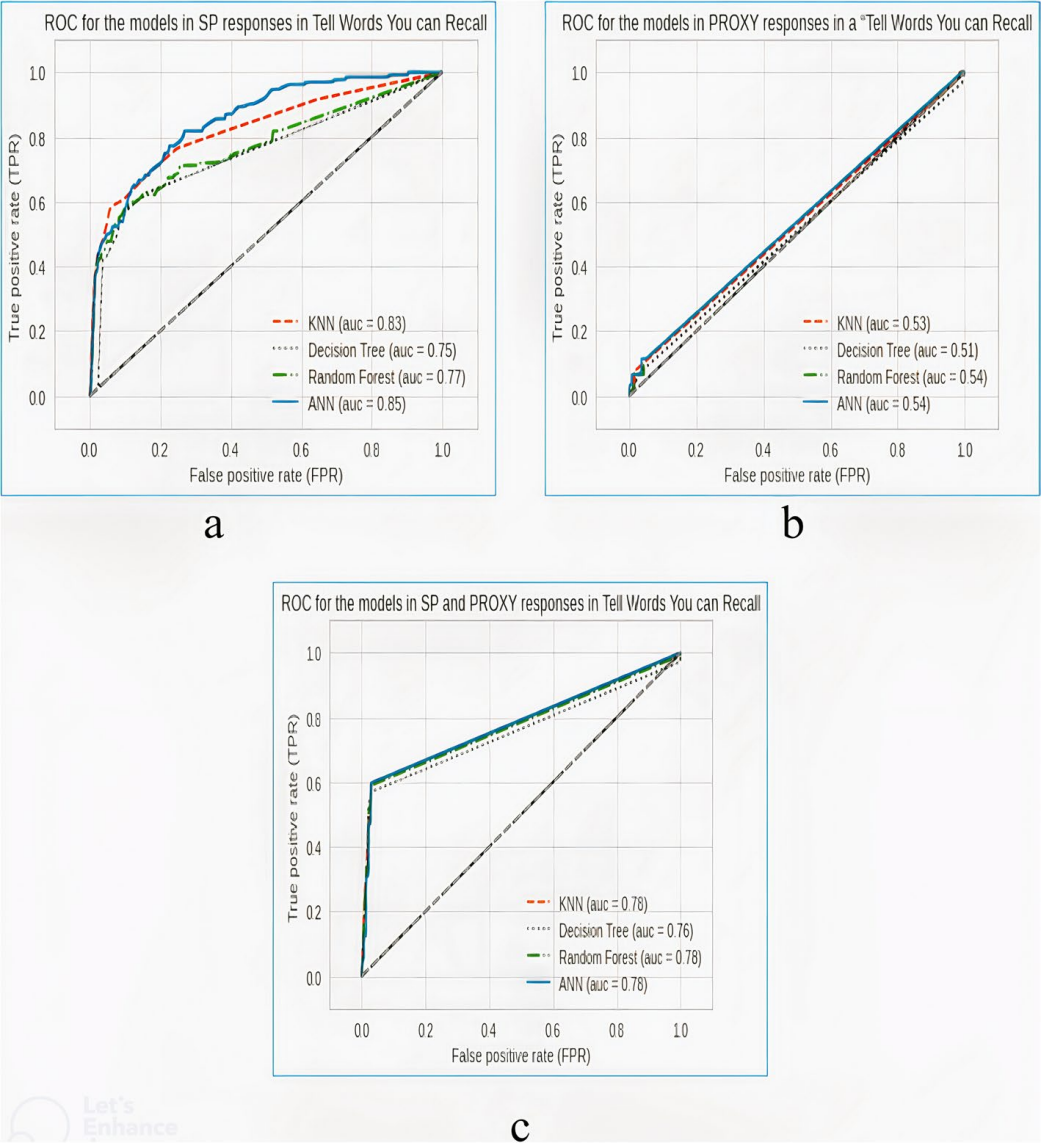
Receiver’s operating characteristics curve obtained from the models in a “word-delay” task based on **a** SP’s responses, **b** proxy’s responses, and **c** a combination of the SP’s and proxy’s responses

Figure 3 shows the receiver’s operating characteristics curves obtained from the models in experiments scenarios 2A, 2B, and 2C.

#### Experiments scenario 3: using “immediate-word-recall” feature

This scenario of experiments focuses on using the “immediate-word-recall” feature in predicting dementia. Each section of the NHATS data that specify the responses of SP and proxy’s responses to the “immediate-word-recall” cognitive assessment was extracted and split into a training set (70%) and a test set (30%). Each of the four models (KNN, RF DT, and ANN) was trained on the training set, and dementia prediction was done on the test.

- *Experiment scenario 3A:* This experiment was carried out using only the SP’s responses to an “immediate-word-recall” cognitive assessment task to model and predict dementia.
- *Experiment scenario 3B*: This experiment was carried out using only the proxy’s responses to an “immediate-word-recall” cognitive assessment task to model and predict dementia.
- *Experiment scenario 3C:* This experiment was carried out using the combination of the SP’s and proxy’s responses to an “immediate-word-recall” cognitive assessment task to model and predict dementia.

For these experiments, Table 3 shows the models’ performances in terms of accuracy, cross-validation accuracy, precision, F1 score, sensitivity, and specificity.

**Fig. 4:**
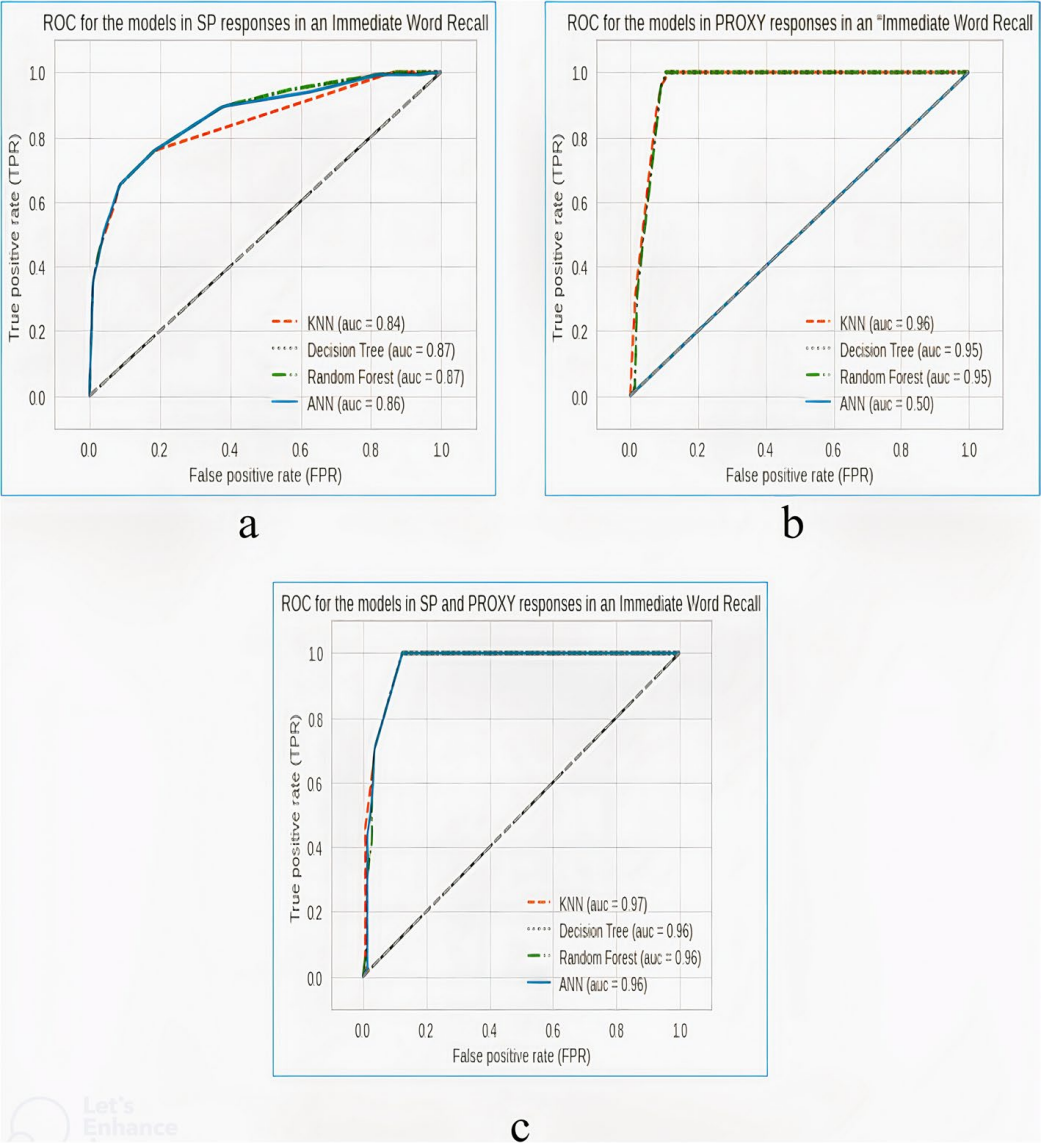
Receiver’s operating characteristics curve obtained from the models in the “immediate-word-recall” task based on **a** SP’s responses, **b** proxy’s responses, and **c** a combination of the SP’s and proxy’s responses

Figure 4 shows the receiver’s operating characteristics curves obtained from the models in experiments scenario 3A, 3B, and 3C.

### Model performance in the experiments scenario 1

In experiment scenario 1A, it was discovered that sensitivity to the dementia category is low, with the best performing model (RF) having a sensitivity of 0.38. This implies that the use of SP’s responses in the word-delay task cannot be solely used in predicting dementia.

Also, in experiment scenario 1B, sensitivity to the dementia category is extremely low, with the best-performing model in this experiment being KNN, having a sensitivity of 0.11. This also implies that proxies’ responses in the word-delay task cannot be solely used in predicting dementia.

In experiment scenario 1C, however, it was discovered that sensitivity to the dementia category sharply increased for all the models as compared to the two previous experiments, which only involved either the proxies’ responses or SP’s responses. In this experiment, the highest performing models in terms of sensitivity to the dementia category include KNN, RF, and ANN, all with 0.60 sensitivity and having the same accuracy of 78.0%. This implies that combining the responses of the SP and the proxies is critical in predicting dementia cases in a word-delay task. The combination of both responses showed the effects of improving the performances of the predictive models used.

### Model performance in the experiments scenario 2

In experiment scenario 2A, sensitivity to the dementia category is low, with the best performing model (ANN) having a sensitivity of 0.44. This implies that the use of SP’s responses in the “tell-words-you-can-recall” task cannot be exclusively used in predicting dementia.

In experiment scenario 2B, it was discovered that sensitivity to the dementia category sharply dropped for all the models compared to all the previous experiments with the best-performing model in this experiment being RF having a sensitivity of 0.10. This simply implies that proxies’ responses in the “tell-words-you-can-recall” task cannot be solely used in predicting dementia cases.

In experiment scenario 2C, sensitivity to the dementia category stands at 0.60 for the KNN model. In this scenario, this particular experiment also proves that combining the responses of the SP and the proxies is critical in predicting dementia cases.

### Model performance in the experiments scenario 3

In experiment scenario 3A, sensitivity to the dementia category is low, with the best-performing model (ANN) having a sensitivity of 0.37. This implies that the use of SP’s responses in the immediate-word-recall task cannot be exclusively used in predicting dementia.

In experiment scenario 3B, it was discovered that sensitivity to the dementia category sharply increased for all the models compared to all the previous experiments with the best-performing models in this experiment being DT and RF having the same sensitivity of 0.98, and other models with the sensitivities of 0.97 and 0.89 for KNN and ANN respectively. This simply implies that the use of proxies’ responses in the immediate-word-recall task can be efficiently used in predicting dementia cases.

Likewise, it was discovered in experiment scenario 3C that sensitivity to the dementia category increased to 1.00 for all the models. Though the use of proxy responses has proven to be more beneficial than those of SP, this particular experiment proves that combining the responses of the SP and the proxies is strongly critical in the prediction of dementia cases. It, therefore, implies that the use of both SP and proxy responses in the immediate-word-recall task has the effect of further improving the performances of the predictive models used.

## Discussion

### Interpretation

In this study, nine distinct experiments were conducted to determine which responses (either SP’s or proxy’s) in the “word-delay,” “tell-words-you-can-recall,” and “immediate-word-recall” tasks are essential in the prediction of dementia cases, and to what extent the combination of these two responses is helpful in the prediction of dementia. It was discovered in all the experiments that the use of combined responses from SP and proxies outperforms the use of only proxies’ responses in the prediction of dementia, which in turn outperforms the use of SP’s responses in this study as depicted in the experiments scenario 1. It can then be concluded that the combination of both the responses obtained from the SP and proxies in the dementia study (based on the NHATS dataset) are clinically useful in the prediction of dementia cases.

As shown in this study, it was further discovered that the use of “word-delay” and “tell-words-you-can-recall" cannot be used in predicting dementia due to their poor performances in all the developed models (as shown in all the experiments in scenarios 1 and 2). However, immediate-word-recall showed its reliability in predicting dementia, as seen in all the experiments in Scenario 3.

Finally, using responses of both SP and proxies in the immediate-word-recall task in experiment scenario 3C outperformed all other experiments. This, therefore, shows the importance of immediate-word-recall in predicting dementia and the efficiency of combining responses of both SP and proxies in the immediate-word-recall task.

This study further establishes that the results obtained were not dependent on the machine learning methods used. Each method performed differently in the experimental scenarios presented.

To check the possible effects of imbalance in the original dataset on the obtained results, we carried out similar experiments on a resampled version of the dataset, which was resampled using the Synthetic Minority Oversampling Technique followed by the Tomek Link technique (SMOTE-Tomek) algorithm. In these supplementary experiments, the same trend reported in this study was also discovered, in that the use of immediate-word-recall shows its reliability in predicting dementia. Likewise, the efficiency of combining responses of both SP and proxies in the immediate-word-recall cognitive assessment task was discovered. The link to the supplementary experiments can be found at https://doi.org/10.6084/m9.figshare.16964662 [33].

### Strengths and limitations

Based on our knowledge, this study appears to be the first study to investigate the use of machine learning techniques for predicting dementia with cognitive features on the NHATS dataset. This paper mainly reports the predictive performances of different cognitive assessment tasks in the prediction of dementia based on the performance of the machine learning models trained on them.

This study is constrained by some methodological choices. The primary focus of this study is on analyzing dementia prediction based on only memory recall test data while other cognitive assessment tests that could be useful in the prediction of dementia were not considered. Informative features from such tests could also be reliably used in dementia prediction.

For simplicity, this study tested some traditional machine learning algorithms, although more complex algorithms (ensembles) could have been considered in building the predictive models from the dataset of interest.

### Future directions

Since this study attempted to focus on the use of word recall, a memory recall test aspect of the cognitive assessment tests to predict dementia, some other attempts could be imagined. First, there are other well-known assessment methods like the Clock Drawing Test [34], short-form and long-form Informant Questionnaire on Cognitive Decline in the Elderly (IQCODE) [35, 36], Memory Impairment Screen [37], Montreal Cognitive Assessment (MoCA) [38], Free and Cued Selective Reminding [39], and Mental Scale Questionnaire [39], among others, that could be considered for developing dementia predictive models. Second, studying the use of ML algorithms on the combined features/data extracted from these cognitive assessment tests could be considered in future work. Third, while this study is not dependent on the choice of the ML algorithms that were used, it could be necessary to consider ensemble methods to predict dementia based on word recall (and possibly other cognitive assessment tests), because they have been noted in the literature to improve predictive models’ performances in some other problem domains [40–43].

## Conclusions

In this study, data on two types of respondents (sample persons and proxies) were considered separately and then combined together for the prediction of dementia. The data from our study suggest that the use of the combination of both the responses obtained from the sample persons and proxies in the dementia study is clinically useful in the prediction of cases of dementia. The use of “word delay” and “tell words you can recall" performed poorly in all the developed models indicating weak potential in predicting cases of dementia. The use of immediate word recall showed reliability in predicting dementia. Finally, results in this study showed the strong potential of immediate word recall cognitive assessment in the prediction of dementia and the efficiency of combining responses of both sample persons and proxies in the immediate word recall cognitive assessment task, for dementia prediction.

## Appendix

### Feature list details

The experiments in this study were further divided into four different categories:

(i) For “Word Delay”, a total 20 features were obtained by combinining,

“Delayed Word Recall:” (“cg8wrdsdcal1”, “cg8wrdsdcal2”, “cg8wrdsdcal3”, “cg8wrdsdcal4”, “cg8wrdsdcal5”, “cg8wrdsdcal6”, “cg8wrdsdcal7”, “cg8wrdsdcal8”, “cg8wrdsdcal9”, “cg8wrdsdcal10”)

AND

“Word Delayed Recall:” (“cg8dwrd1dly”, “cg8dwrd2dly”, “cg8dwrd3dly”, “cg8dwrd4dly”, “cg8dwrd5dly”, “cg8dwrd6dly”, “cg8dwrd7dly”, “cg8dwrd8dly”, “cg8dwrd9dly”, “cg8dwrd10dly”)

This set of derived variables reflects the number of people who recalled the 1st word in the list, the number who recalled the 2nd word in the list, etc.

(ii) The “Tell Words you can Recall” has 10 features (“cg8wrdsrcal1”, “cg8wrdsrcal2”, “cg8wrdsrcal3”, “cg8wrdsrcal4”, “cg8wrdsrcal5”, “cg8wrdsrcal6”, “cg8wrdsrcal7”, “cg8wrdsrcal8”, “cg8wrdsrcal9”, “cg8wrdsrcal10”).

This reflects the number of people who recalled at least 1 word, at least 2 words, etc. up to 10 words by the word recalled (word 1, 2, 3, etc.). For example, cg8wrdsrcal9 (the variable that reflects results for persons who remembered at least 9 words on immediate word recall) which can be interpreted as “set of those who recalled at least nine words”.

(iii) The “Immediate Word Recall” has only one feature (“cg8dwrdimmrc”).

This feature has 10 categories ranging from 0 to 9, based on the numbers of words the SP can recall from the list of words given.

(iv) The last category was developed using PCA with n_components of 12.

More description of the features in the dataset can be found in Kasper and Freedman [32].

## Data Availability

The data that support the findings of this study are available from National Health and Aging Trends Study (NHATS), but restrictions apply to the availability of these data, which were used under license for the current study, and so are not publicly available. Data are however available with permission of the National Health and Aging Trends Study at https://nhats.org/researcher/data-access/public-use-files. Also, the data generated during the current study are available in the figshare repository, https://doi.org/10.6084/m9.figshare.16817506.v3.
